# Semantic and Geographical Analysis of COVID-19 Trials Reveals a Fragmented Clinical Research Landscape Likely to Impair Informativeness

**DOI:** 10.3389/fmed.2020.00367

**Published:** 2020-06-29

**Authors:** Giulia Tini, Bruno Achutti Duso, Federica Bellerba, Federica Corso, Sara Gandini, Saverio Minucci, Pier Giuseppe Pelicci, Luca Mazzarella

**Affiliations:** ^1^Department of Experimental Oncology, IEO European Institute of Oncology IRCSS, Milan, Italy; ^2^Department of Hemato-Oncology, University of Milan, Milan, Italy; ^3^Division of Early Drug Development for Innovative Therapies, IEO European Institute of Oncology IRCCS, Milan, Italy

**Keywords:** COVID, trial, geography, endpoint, design

## Abstract

**Background:** The unprecedented impact of the COVID-19 pandemic on modern society has ignited a “gold rush” for effective treatment and diagnostic strategies, with a significant diversion of economic, scientific, and human resources toward dedicated clinical research. We aimed to describe trends in this rapidly changing landscape to inform adequate resource allocation.

**Methods:** We developed an online repository (COVID Trial Monitor) to analyze in real time the growth rate, geographical distribution, and characteristics of COVID-19 related trials. We defined structured semantic ontologies with controlled vocabularies to categorize trial interventions, study endpoints, and study designs. Analyses are publicly available at https://bioinfo.ieo.it/shiny/app/CovidCT.

**Results:** We observe a clear prevalence of monocentric trials with highly heterogeneous endpoints and a significant disconnect between geographic distribution and disease prevalence, implying that most countries would need to recruit unrealistic percentages of their total prevalent cases to fulfill enrolment.

**Conclusions:** This geographically and methodologically incoherent growth casts doubts on the actual feasibility of locally reaching target sample sizes and the probability of most of these trials providing reliable and transferable results. We call for the harmonization of clinical trial design criteria for COVID-19 and the increased use of larger master protocols incorporating elements of adaptive designs. COVID Trial Monitor identifies critical issues in current COVID-19-related clinical research and represents a useful resource with which researchers and policymakers can improve the quality and efficiency of related trials.

## Introduction

Standard and effective approaches for COVID-19 prevention and treatment are not available to date, despite the magnitude of the pandemic and the similarities with the past coronavirus-associated diseases SARS and MERS (1). So far, initial trials with antivirals or other potentially effective drugs such as chloroquine have not yet clearly demonstrated superior efficacy over alternative treatments (2–4), and the disease remains associated with devastating morbidity and mortality. A wide variety of intervention strategies have been proposed, aiming at different mechanisms (viral or host processes), disease stages (early, advanced, or prevention), and intervention modalities (medical or non-medical).

As COVID-19-devoted resources grow, quantifying the potential impact of COVID-19 trials becomes a relevant matter for global and national health policies. However, quality research on clinical trials is rendered difficult by the lack of a standardized definition of trial parameters. Data reporting in trial repositories is notoriously plagued by internal inconsistencies, especially for “free text” fields that contain key information like inclusion criteria or study endpoints (5). General medical ontologies like MeSH terms provide an all-encompassing framework but may be inadequate to capture relevant distinctions for specific fields; COVID-related terms were only introduced in late March, and their use is only recommended and not mandatory for trial definition.

In the present work, we defined structured semantic ontologies with controlled vocabularies to categorize trial interventions, study endpoints, and study designs, and we conducted an analysis of the growth rate, geographical distribution, and trial characteristics of COVID-19-related trials, highlighting a number of relevant features that may impair the possibility of obtaining reliable and transferable results within the current framework. We formulate proposals for more rational trial designs against this rapidly changing landscape.

## Results

### Global Growth Rate

**Figure 1:**
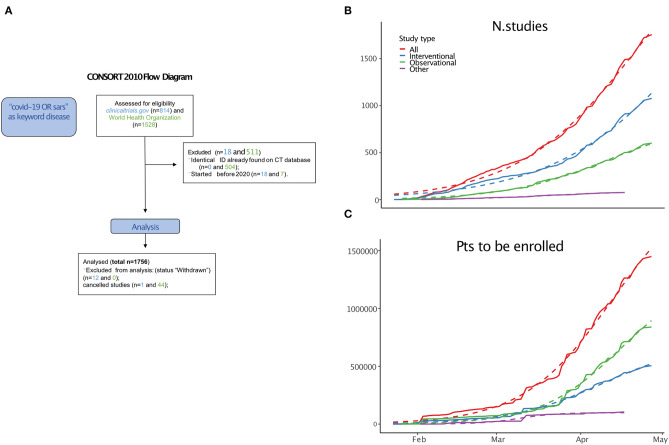
**(A)** CONSORT diagram. **(B)** Cumulative growth of trials. **(C)** Projected enrolled patients (thousands). See Supplementary Data for equation parameters.

We identified 1,756 relevant studies (including interventional, observational, and other) combining entries from the WHO and ClinicalTrials.gov databases (Figure 1A).

From 23 January, 2020 (the date of the first study posted), the cumulative increase in the number of studies (Figure 1B) and the projected enrolled patients (Figure 1C) have been growing logistically.

We analyzed the funding source on the 519 interventional trials from ClinicalTrials.gov for which this information was available (Table 1) and found that a high percentage (397, 76.49%) are funded by public agencies, 62 (11.95%) by industries, and 52 (10.02%) by private–public collaborations. Comparison with a disease of comparable magnitude like influenza or cancer shows how this ratio of industry vs. non-industry is highly unusual (influenza 15/47, 31.91%, *p* = 7.75 × 10^−5^; cancer 735/3167, 23.21%, *p* < 2.2 × 10^−16^); instead, no significant differences were found for private–public collaborations (influenza, 6%, cancer 12%, *p*-values are non-significant).

**Table 1:** Sources of funding.

**Study type**	**Interventional**	**Observational**	**TOTAL**
Other	397	241	638
Industry	62	11	73
Oth/Ind	52	7	59
OTH/NIH	3	0	3
NIH	2	5	7
U.S.Fed	2	0	2
U.S.Fed/OTH	1	0	1
Total	519	264	783

*Limited to clinicalTrials.gov. “OTH/IND” represent studies that were funded by a collaboration between industry and public sources, “OTH/NIH” studies were funded by public sources and NIH, and “U.S.FED/OTH” are those funded by public sources and the US Fed*.

For subsequent analyses, we focused on interventional trials (*n* = 1078), although data have been collected for all trials and are available in Supplementary Table 1.

### Geographical Distribution

**Figure 2:**
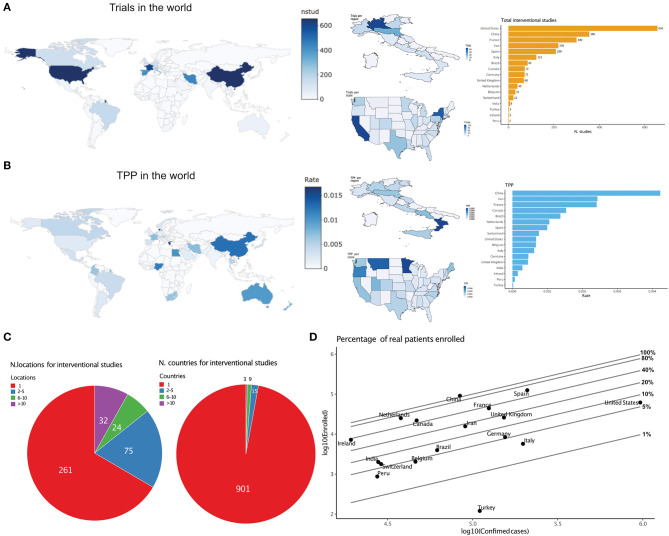
Geographical distribution. **(A)** Total trials by nation (left) and by region or state in Italy and the USA (middle), and bar graph of the first 17 nations. Countries with <1000 confirmed cases are not reported. **(B)** Trials Per Patient (TPP) by nation (left) and by region or state in Italy and USA (middle), and bar graph of the first 17 nations. Countries with <1000 confirmed cases are not reported. **(C)** Distribution of trials with 1, 2–5, 6–10 or >10 locations (left) or states (right). **(D)** Relationship between projected national enrolment and current cumulative confirmed cases by state. Reference lines project the percentage of all confirmed cases to be enrolled. If a point sits on the 10% line, it means that 10% of all confirmed cases must be enrolled in a trial to satisfy enrolment projections for that country.

Trials were opened in 63 different countries. At the national level, the United States was the nation with the highest number of trials, followed by China (Figure 2A).

We calculated a simple “trials per patient” index (TPP) for each country by dividing the number of available trials by the number of cumulative COVID-19 cases in the country. This index may help to gauge the feasibility/accessibility trade-off for trials ongoing in that country: a high index (=many trials relative to the patient population) suggests unrealistic enrolment needs (in other words, it is unlikely that all trials will fulfill the required enrolment), whereas a low index suggests low access to experimental treatments. Trials per patient (TPP) were unevenly distributed among and within nations (Figure 2B), with a Gini coefficient equal to 0.76. Of the 392 trials with available location information, the vast majority were monocentric (261, 66.58%), while 131 were multicentric. Of those, just 32 were opened in more than 10 locations (Figure 2C).

The correlation between the cumulative projected patient enrolment and the actual case prevalence in each state was poor (Pearson=0.37). With current case prevalence, most countries would need to recruit extremely high and possibly unrealistic percentages of their total prevalent cases to fulfill enrolment (Figure 2D).

### Characteristics of Interventional Trials and Types of Intervention

**Figure 3:**
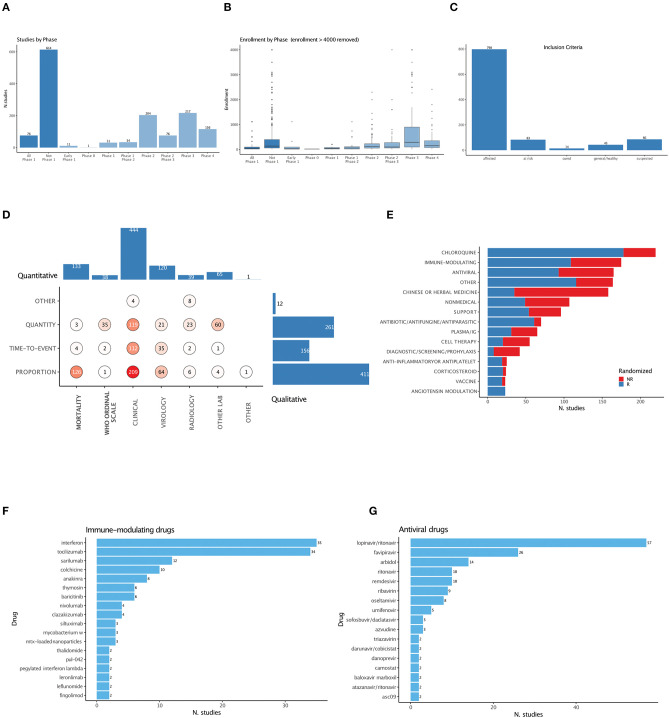
Trial features. **(A)** Number of trials by phase. **(B)** Cumulative planned enrolled patients by phase. One trial with >4000 enrolments was removed from the graph. **(C)** Distribution of inclusion criteria. **(D)** Distribution of primary study endpoints. “Hard” endpoints, defined as including mortality, are in bold. **(E)** Distribution of intervention categories and use of randomized designs. **(F)** Breakdown of immune-modulating drugs. **(G)** Breakdown of antiviral drugs.

Early-phase studies (phase 1 and 1-2) were under-represented in both numbers and patients (Figures 3A,B). To better describe and capture the semantic heterogeneity of trial characteristics, we defined ontologies with controlled vocabularies for interventions, study designs (Supplementary Table 2), inclusion criteria (Supplementary Table 3), and study endpoints (Supplementary Table 4).

Among trials aimed at active treatment, a significant share (86/1078) do not require PCR-confirmed diagnosis as inclusion criteria (“suspected,” Figure 3C). Primary endpoints are qualitatively (clinical or virological, radiological, or other laboratory variables) and quantitatively (411 proportion, 156 time-to-event, 261 quantity) heterogeneous; “hard” endpoints containing mortality either use the incommensurable quantitative WHO ordinal scale or proportional measures (Figure 3D).

We categorized all interventional treatments under 15 terms. Randomization is common but not prevalent among most interventions (Figure 3E); Chloroquine, immune-modulating agents (expanded in Figure 3F), and antivirals (expanded in Figure 3G) are the most investigated, with 220, 175, and 165 studies, respectively.

## Discussion

We present quantitative, updated, and semantically organized measures of COVID-19-related trials. We highlight a number of peculiar characteristics of this clinical research landscape: extremely rapid growth, substantial geographical and methodological incoherence, an unusual funding pattern, prevalence of monocentric trials, and extreme heterogeneity in the interventions tested. These characteristics are unprecedented in the history of clinical research, a consideration that prevents meaningful comparison with the research landscapes of other prior major outbreaks.

The main limitation of our analysis is represented by the heterogeneity in terms of quality and quantity of the available information. The source databases often use non-overlapping trial categorization methods, and many of the records have missing, misspelled, or imprecise wording, potentially causing relevant selection biases. We attempted to mitigate these by forcing information through controlled vocabularies, a procedure that may result in loss of information.

We argue that several of the planned trials are unlikely to provide high-quality results for the following reasons.

First and foremost, the unrealistic percentages of total prevalent cases needed to fulfill planned enrollment at the national level imply that several trials are unlikely to reach target sample sizes, with severe loss of statistical power or study termination. This has in fact already been observed with the recently published Remdesivir trial in China, which failed to complete enrolment, leading to conflicting interpretations (6).

Geographical fragmentation will magnify local and study-specific confounding in demographics, comorbidities, and the availability of healthcare resources, which are known to impact COVID-19 outcome heavily (7–9). Variegated endpoints and inclusion criteria will inhibit the possibility of adequately comparing and meta-analyzing treatments across trials. Proper dose-finding trials are scarce, giving rise to a risk of under- or over-treating patients and of underestimating potentially risky drug-interactions. Finally, the scientific soundness of classical randomized designs in a scenario where the control arm may be rapidly changing (10) is ethically and methodologically questionable.

Our analysis provides quantitative grounds for concerns raised in the early days of the COVID-19 pandemic in commentaries (11–13) that highlighted the difficulties of striking a balance between the need to conduct sound clinical research and the need to take rapid action. This observed disordered growth in clinical research is perhaps expected given the unprecedented medical and socio-economic impact of the COVID-19 pandemic and the absence of homogeneous and clear-cut guidelines on key aspects of COVID-19-related clinical research, such as what should be considered the gold standard for control arms or the primary endpoints for drug approval. However, we note that the scientific community should prepare the ground for a more ordered development, especially in light of the expected persistence of SARS-CoV2 and the likely emergence of other coronavirus-mediated diseases in the long run.

A potential solution for some of the above issues is to favor the adoption of adaptive trial design features (inclusion of predefined toxicity/efficacy stopping rules, biomarker-adjusted enrolment, etc.) and the inclusion of multiple phases, interventions, and patient groups under the same regulatory framework, using the so-called “master protocol” model (14). Advantages of this model include (i) the possibility of comparing the efficacy of multiple interventions against a single, well-standardized control arm, (ii) the possibility of comparing across multiple treatments, particularly relevant in a scenario where time bias is likely to play a major role: mortality is likely to be subject to time-dependent variables such as the ICU occupancy ratio or physician experience acquired, (iii) the possibility of skewing enrolment into more effective/less toxic arms as new data are accumulated, (iv) the possibility of introducing novel treatment arms or stratification biomarkers as these are identified in preclinical or translational studies, and (v) the possibility of collecting samples for translational studies under a unified and homogeneous framework, increasing their informativeness. We identified 18 trials with declared adaptive features in their designs (Supplementary Table 5), among which the most notable is the large SOLIDARITY trial promoted by the WHO to test four treatment options (Remdesivir; Lopinavir/Ritonavir; Lopinavir/Ritonavir with Interferon beta-1a; Chloroquine or Hydroxychloroquine) against standard of care.

Master protocols are themselves subject to specific biases, in particular the need to adjust for multiple hypothesis testing (14, 15), and often require sophisticated monitoring and logistics that can only be accomplished within large organizations. This calls for stronger interaction between stakeholders like pharma companies, regulatory bodies, funding entities, and patient organizations. In the present rapidly changing scenario, such frameworks may be of particular utility to efficiently discard non-viable hypotheses and prioritize treatment that deserves proper testing on larger scales. Experience gained in some fields where master protocols are increasingly adopted, such as oncology (16, 17), may inform trial design.

## Methods

### Databases

Data were downloaded from ClinicalTrials.gov and the WHO International Clinical Trials Registry Platform (ICTRP https://www.who.int/ictrp/en/) on April 11 and 27.

Data for COVID cases by country and for US states were downloaded from the Johns Hopkins Data Repository (https://github.com/CSSEGISandData/COVID-19) and for Italian regions from Presidenza del Consiglio dei Ministri–Dipartimento della Protezione Civile (https://github.com/pcm-dpc/COVID-19) on April 27.

Details on ontology definition, geographical analyses, and statistical analyses are discussed in the Supplementary Methods.

## Data Availability Statement

All datasets generated for this study are included in the article/Supplementary Material.

## Author Contributions

GT and LM: collected data, performed analyses, and wrote the manuscript. BD, FB, and FC: performed analyses. SG, SM, and PP: wrote the manuscript. All authors contributed to the article and approved the submitted version.

## Conflict of Interest

The authors declare that the research was conducted in the absence of any commercial or financial relationships that could be construed as a potential conflict of interest.
