# Supplementary material for: Semantic and Geographical Analysis of COVID-19 Trials Reveals a Fragmented Clinical Research Landscape Likely to Impair Informativeness
Source: Front Med (Lausanne). 2020 Jun 29;7:367. doi: 10.3389/fmed.2020.00367 (PMC7336807; doi:10.3389/fmed.2020.00367)
Supplement: Supplementary file 6 [file Data_Sheet_1.docx]

## Supplementary methods

**Definition of trial ontologies**

We defined the following internal algorithm for trial feature classification, for each of the three curated domains (inclusion criteria, interventions, endpoints)

1. all records at April 10^th^ were revised by the panel to identify the features deemed most relevant for the infomativeness for Covid management and the classification rules (see below)

2. all records were re-classified using the established rules by 2 members of the panel, composed by statisticians (FB, FC, GT) and physicians with experience in trial design (LM, BD)

3. disagreement between the 2 reviewers was assessed by a third internal reviewer

4. Last, the same process (steps 2-3) were repeated to update the database with records as of April 27^th^. Records will be updated at least on a weekly based to maintain this as a live resource available at <https://bioinfo.ieo.it/shiny/app/CovidCT>

For **inclusion criteria**, we discriminated based on the definition of the disease setting into:

Affected: subjects who require SARS-CoV2 infection confirmation, usually by PCR

Suspected: subjects with symptoms of Covid-like pneumonia but not necessarily PCR-confirmed

Cured: subjects who require a negativeSARS-CoV2 after an initial positive diagnosis

At risk: subjects without confirmed SARS-CoV2 infection, enrolled on the basis of specific risk factors (healthcare workers, cancer patients, epidemiological exposure, etc)

General/healthy: subjects without confirmed SARS-CoV2 and no specific risk factor

We also developed a hierarchical characterization for some terms to account for multiple inclusion criteria. For example, if a study allows for both affected and suspected cases but does not *require* infection confirmation, the “suspected” category would be chosen

For **interventions**, we classified them in 2 layers. The lower layer is the actual drug, for which we tried to homogenize the nomenclature where possible, trying to always use the chemical name (for instance, arbidol or arbirdol or umifenovir would be homogeneized to umifenovir). The upper layer is the drug category, that was defined based on similarities in the general mechanism of action. In just one case, a group corresponds almost entirely with a single drug and its derivative (chloroquine-hydroxychloroquine), given the relevance of this drug for current Covid research. When multiple interventions were present in the same study, administered either in combination or in different arms, they were independently classified and each calculated individually in figure 1F

For **endpoints**, we defined two “axes”:

-quantitative: the type of statistics used to define the endpoint, as continuous (e.g. symptom scales or continuous laboratory values), proportion (e.g. percentage of mortality after defined time point) or time-to-event (e.g. time to ventilator weaning)

-qualitative: the nature of the readout used to assess the endpoint. The first dichotomy was between endpoints of therapeutic efficacy vs other (prevention, diagnosis)

For therapeutic efficacy endpoints, as overall survival is often considered as the strongest endpoint, being devoid of biases due to subjectivity or experimental error, we categorized them as:

-“hard” when included mortality. We further characterized these into those adopting the WHO symptomatic scale (WHO W) and all others (mortality M)

-“surrogate” when not explicitly including mortality. These were classified as based on viral tests (e.g. percentage of PCR-negative subjects), radiological tests (e.g. variation in radiological scores), serological tests (e.g. percentage of seroconverted subjects), other laboratory tests (e.g. levels of inflammatory markers) clinical (e.g. symptom scales not explicitly including mortality)

For non-efficacy endpoints, we defined preventive (e.g. percentage of PCR-positive subjects in a prevention study), diagnostic (e.g. sensitivity of a laboratory test) or other (e.g. scores on psychology scales)

**Number of trials and Enrollment**

The growth of the cumulative number of trials during the last months was fitted with a logistic growth in the form $y=\frac{\alpha}{(1+e^{-\frac{(x-\beta)}{\gamma}})}$, to respect the classic epidemic growth, which grows until it reaches a plateau. Models were designed for the overall number of studies and separately for interventional, Observational and Other study types. The functions nls and SSlogis from R were used to this aim. As starting point of the analysis was chosen January 20th, day of the first study posted. Statistical significance was assessed by coefficient pvalues<0.05.

Similarly, logistic growths for the number of cumulative enrolled patients were fitted for the overall number of patients, and for those enrolled into interventional, observational and other studies.

To obtain convergence, in the case of “observational” and “other” the number of iterations was increased from 50 to 500.

**Equations for figures 1B:**

**Logistic models** $y=\frac{\alpha}{(1+e^{-\frac{x-\beta}{\gamma}})}$**for n. studies in time (y=n.trials, x=dates) fig 1B top**

- “All”: $y=\frac{3340}{(1+e^{-\frac{x-1.838\times{10}^{4}}{22.70}})}$ (pvalues <$2\times{10}^{-16}$)
- “Interventional”: $y=\frac{3578.610}{(1+e^{-\frac{x-1.8399\times{10}^{4}}{26.607}})}$ (pvalues: alpha=$1.27\times{10}^{-5}$, others<$2\times{10}^{-16}$)
- “Observational”: $y=\frac{846.3}{(1+e^{-\frac{x-1.836\times{10}^{4}}{18.63}})}$ (pvalues <$2\times{10}^{-16}$)
- “Other”: $y=\frac{90.30}{(1+e^{-\frac{x-1.834\times{10}^{4}}{16.01}})}$ (pvalues <$2\times{10}^{-16}$)

**Logistic models** $y=\frac{\alpha}{(1+e^{-\frac{x-\beta}{\gamma}})}$**for enrolled patients in time (y=enrollment, x=dates) fig 1B low**

- “All”: $y=\frac{2.189\times{10}^{6}}{(1+e^{-\frac{x-1.837\times{10}^{4}}{16.70}})}$ (pvalues <$2\times{10}^{-16}$)
- “Interventional”: $y=\frac{7.017\times{10}^{5}}{(1+e^{-\frac{x-1.836\times{10}^{4}}{16.93}})}$ (pvalues <$2\times{10}^{-16}$)
- “Observational”: $y=\frac{1.306\times{10}^{6}}{(1+e^{-\frac{x-1.837\times{10}^{4}}{14.65}})}$ (pvalues <$2\times{10}^{-16}$)
- “Other”: $y=\frac{9.869\times{10}^{4}}{(1+e^{-\frac{x-1.833\times{10}^{4}}{7.058}})}$ (pvalues: gamma: $3.06\times{10}^{-10}$), others <$2\times{10}^{-16}$)

**Funding source**

We compared the amounts of Covid19 studies funded by industries and public sources with their correspondent cases recovered from influenza and cancer. To this aim, we performed Fisher exact test: pvalues below 0.05 were used to assess statistical significance. Clinical trials for the other diseases were downloaded by clinicaltrials.gov. To perform a more balanced analysis, only studies registered in 2019 and 2020 were considered.

Despite we limited our analysis to clinicaltrials.gov, most of the trials registered by WHO, especially those registered in China has as primary sponsor a hospital, in line and confirming our analysis above.

**Geographical distribution**

We considered the location of the studies, when available to explore the geographical distribution of studies and their effect on Covid19 patients. We defined as multicentric those studies that were opened in more than one site.

For each country, we computed the ratio of the number of trials and the number of patients: we called this index “TPP” (trials per patients). To understand the level of inequality of TPP among countries with at least 1000 confirmed COVID-19 cases, we used the Gini index (computed with ineq R package): this index measures 0 when the distribution is homogeneous, 1 when it is totally unequal. We observed inequalities also by comparing the total enrollment in a nation (sum of all the enrollment, enrollment of multicentric studies was equally divided by all the sites) to different percentages of real number of patients.

To obtain a higher detail of the TPP, we computed its value also for Italian regions and US states. To this aim, we used data coming only from clinicaltrias.gov, which provides the precise location of the trials. Geographical coordinates were obtained with the R package tmaptools, and each location was then associated to a state/region. Data of Covid19 cases in Italy were downloaded from <https://github.com/pcm-dpc/COVID-19/tree/master/dati-regioni>, those for USA from <https://github.com/CSSEGISandData/COVID-19/tree/master/csse_covid_19_data/>

All the statistical analyses described were performed using R v 3.6.0
